# Modeling defects and plasticity in MgSiO_3_ post-perovskite: Part 1—generalized stacking faults

**DOI:** 10.1007/s00269-015-0762-9

**Published:** 2015-07-18

**Authors:** Alexandra M. Goryaeva, Philippe Carrez, Patrick Cordier

**Affiliations:** Unité Matériaux et Transformations - UMR CNRS 8207 - Bat C6, Université Lille 1, 59655 Villeneuve d’Ascq Cedex, France

**Keywords:** MgSiO_3_ post-perovskite, High pressure, Generalized stacking faults

## Abstract

In this work, we examine the transferability of a pairwise potential model (derived for MgSiO_3_ perovskite) to accurately compute the excess energies of the generalized stacking faults (GSF, also called *γ*-surfaces) in MgSiO_3_ post-perovskite. All calculations have been performed at 120 GPa, a pressure relevant to the D″ layer. Taking into account an important aspect of crystal chemistry for complex materials, we consider in detail all possible locations of slip planes in the post-perovskite structure. The *γ*-surface calculations emphasize the easiness of glide of slip systems with the smallest shear vector [100] and of the [001](010) slip system. Our results are in agreement with previous ab initio calculations. This validates the use the chosen potential model for further full atomistic modeling of dislocations in MgSiO_3_ post-perovskite.

## Introduction

In 2004, the discovery of the so-called “last mantle phase transition” immediately became promising to shed light on the puzzling properties of the D″ layer and to provide new insights into our understanding of the dynamics of the lowermost part of the Earth’s mantle. The transformation of the magnesium silicate perovskite (bridgmanite) into a denser, so-called post-perovskite phase, was discovered with input from both experiment and theory (Murakami et al. 2004; Oganov and Ono 2004; Tsuchiya et al. 2004a). According to the observations, the magnesium silicate post-perovskite is only stable at high-pressure and high-temperature conditions in excess of 120 GPa and 2000 K corresponding to the lowermost ~150 km of the mantle. Despite uncertainties in both the thermochemical state of the lowermost mantle and the pressure–temperature conditions of the transition (Grocholski et al. 2012), the post-perovskite is likely to be a major component of the lowermost lower mantle (Cobden et al. 2012; Hutko et al. 2008). A major question is whether this phase can explain some of the intriguing properties of the D″ layer, including its marked seismic anisotropy (Su and Dziewonski 1997; Sidorin et al. 1999; Panning and Romanowicz 2004). Indeed, post-perovskite has immediately attracted attention because of its crystal structure. Its orthorhombic unit cell (space group *Cmcm, z* = 4) is very anisotropic, with *a* = 2.456 Å, *b* = 8.042 Å and *c* = 6.093 Å (Murakami et al. 2004). Uncommonly for high-pressure phases, the post-perovskite structure is characterized by the presence of two non-equivalent cationic layers parallel to (010). These layers are formed by Si-octahedra and eightfold coordinated Mg exclusively. The occupied Wyckoff positions are (*4c*) for Mg, (*4a*) for Si, and (*4c*) and (*8f*) for O. Not surprisingly, this crystal structure results in several anisotropic physical properties. From the elastic point of view, the much lower value of the *C*_22_ modulus compared to *C*_11_ and *C*_33_ shows that the post-perovskite phase is much more compressible along the *b*-axis than along the *a* or *c*-axis (Iitaka et al. 2004; Tsuchiya et al. 2004b). Obviously, anisotropic elastic properties (Stackhouse et al. 2005) are an important parameter in interpreting the potential seismic signature of post-perovskite (e.g., Walker et al. 2011). However, other physical properties are also affected. Atomic diffusion of Mg^2+^ and Si^4+^ in post-perovskite is also extremely anisotropic, with almost eight orders of magnitude difference between the fast and slow directions (Ammann et al. 2010). Metadynamics trajectories identified preferential plane sliding involving the formation of stacking faults as the source of an easy pathway for the phase transition (Oganov et al. 2005; see also Zahn 2011 for the role of shear and stacking faults on the transition between the perovskite and post-perovskite structures). This raises the question of the response of the post-perovskite structure to shear. This question has profound implications on the plastic properties of the phase which in turn may determine seismic anisotropy (Nowacki et al. 2013) and affect the flow at the core–mantle boundary (Nakagawa and Tackley 2011).

The calculation of generalized stacking faults (GSF, also called hereafter *γ*-surface) represents a first powerful approach to crystal plasticity (Vitek 1968). By application of a rigid-body shear to a crystal structure followed by excess energy calculation, it is possible to identify easy shear paths. *γ*-Surfaces also represent a key ingredient in dislocation core modeling with the Peierls–Nabarro model. This approach has been applied to post-perovskite by Carrez et al. (2007) where *γ*-lines were calculated for several potential slip systems demonstrating a significant plastic anisotropy on post-perovskite revealed either by the *γ*-surfaces or by the dislocation modeling. However, both calculations were suffering limitations which prevented from fully clarifying the issue of plastic anisotropy in post-perovskite. These limitations are illustrated by the fact that distinct models are found for screw dislocations depending on the glide plane considered (see as an illustration the case of [001] dislocations in Carrez et al. 2007). This is a result of application of the Peierls–Nabarro model which searches for a solution corresponding to planar core spreading exclusively. Solving the issue of the actual (potentially 3D) core structure of screw dislocations (which may eventually constrain plastic anisotropy) requires further modeling techniques. In this series of papers, we propose to apply full atomistic modeling to establish the 3D structure of dislocations cores in post-perovskite without any assumption. However, such an approach requires modeling of large atomic systems which are beyond computational capabilities as long as first-principle calculations are used. Alternatively, atomistic calculations can be performed on large systems containing defects if atomic interactions are calculated from empirical potentials. However, the use of these potentials fitted against equilibrium properties must be validated in case of plasticity studies which involve atomic configurations far from equilibrium (see for instance, Lu et al. 2000; Zimmerman et al. 2000; Godet et al. 2003; Carrez et al. 2008; Ryu et al. 2009).

Numerous theoretical studies have already showed interatomic potential modeling to be very effective for high-pressure and high-temperature simulations of the perovskite phase (Oganov et al. 2000; Ito and Toriumi 2010; Chen et al. 2012; Gouriet et al. 2014; Hirel et al. 2014). In the present work, the ability of this parameterization to reproduce the structure, elastic properties and *γ*-surfaces of the MgSiO_3_ post-perovskite is examined (by comparing with previous calculations based on first principles). Having done that, we propose a detailed investigation of the influence of crystal chemistry on plastic shear properties of post-perovskite. This study is intended to serve as a basis for the full atomistic modeling of dislocations that will follow.

## Computational methods

In this study, we focus on post-perovskite with the pure MgSiO_3_ composition. Indeed, Metsue and Tsuchiya (2013) have shown that the introduction of iron has little effect on the *γ*-surfaces without changing the plastic anisotropy style of this phase. The classical semi-empirical approach is applied for the theoretical study of the MgSiO_3_ post-perovskite. Force field used in the following is based on an interatomic pairwise potential (1) which takes into account long-range and short-range interactions through Coulombic and Buckingham forms, respectively. The short-range interactions include repulsive and attractive van der Waals interactions:

1 $${U_{ij}}\left( {{r_{ij}}} \right) = \frac{{{z_i}{z_j}}}{{{r_{ij}}}} + {b_{ij}}\exp \left(- {\frac{{{r_{ij}}}}{{{\rho_{ij}}}}} \right) - \frac{{{c_{ij}}}}{{r_{ij}^6}},$$

where *r*_*ij*_ corresponds to the distance between ions with charges *z*_*i*_ and *z*_*j*_; *b*_*ij*_, *ρ*_*ij*_ and *c*_*ij*_ are constant parameters describing the short-range interactions. The parameterization used in this work (Table 1) was previously derived by Oganov et al. (2000) for MgSiO_3_ perovskite (bridgmanite).

**Table 1:** Parameterization of the pairwise potentials (from Oganov et al. 2000)

Bond *ij*	*b*_*ij*_ (eV)	*ρ*_*ij*_ (Å)	*c*_*ij*_ (eV·Å^−6^)
Mg–O	1041.435	0.2866	–
Si–O	1137.028	0.2827	–
O–O	2023.8	0.2674	13.83

Molecular static simulations are carried out at *P* = 120 GPa using the program packages GULP (Gale and Rohl 2003) for optimization of ground-state structural parameters and elastic properties, and LAMMPS (Plimpton 1995) for calculation of *γ*-surfaces. Both codes rely on Ewald summation methods for Coulombic interactions. Optimization is performed using a conjugate gradient algorithm with a stopping tolerance for force on atoms of 10^−9^ eV/Å (1.602×10^−1^–18 N). The athermal elastic constants *C*_*ij*_ are calculated as the second derivatives of the energy density with respect to external strain using the procedure fully implemented in GULP code. The orthorhombic symmetry of post-perovskite results in the elastic stiffness tensor containing nine independent *C*_*ij*_ coefficients.

**Fig. 1:**
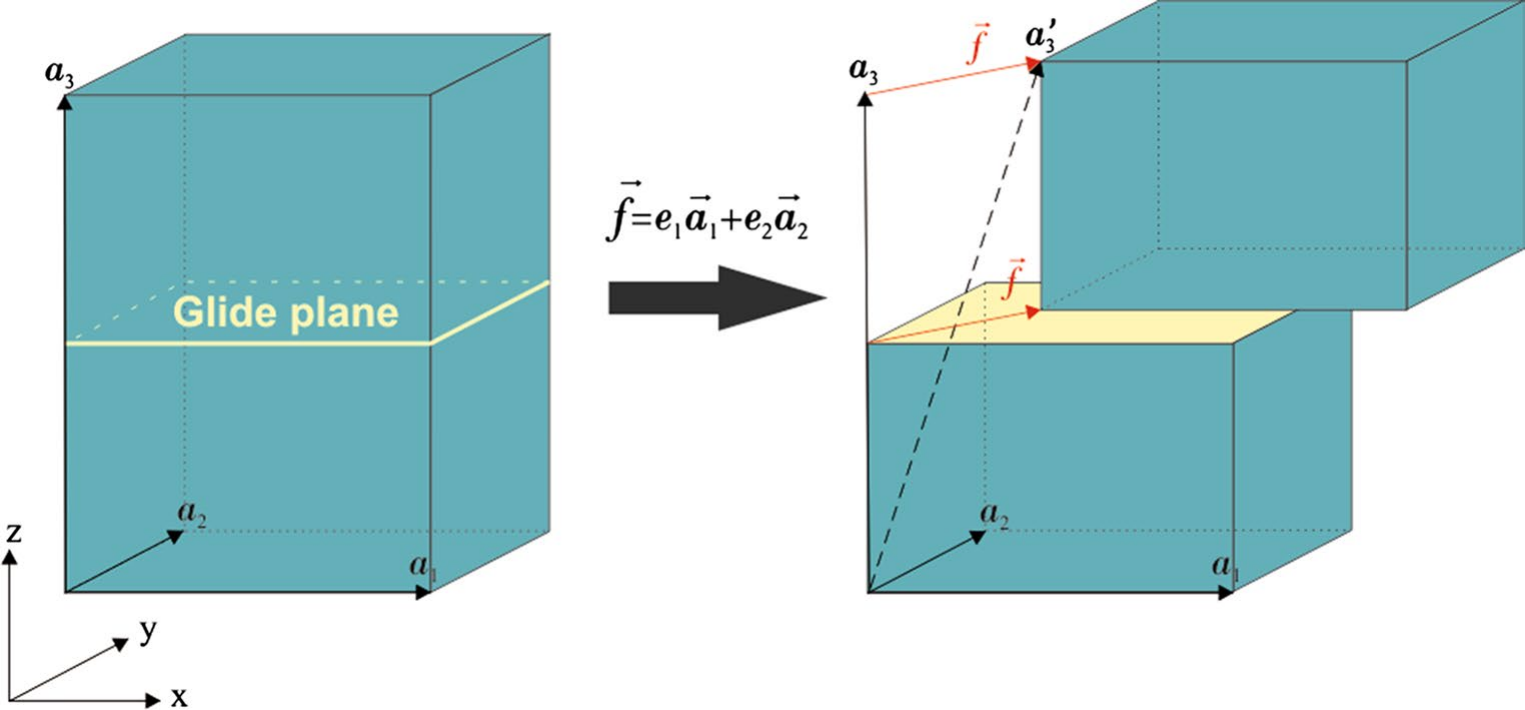
Geometry of atomic systems designed for *γ*-surface calculations. The actual crystallographic orientations used for different (*hkl*) glide planes are reported in the text

For the evaluation of the excess energies associated with a generalized stacking fault, we use fully periodic supercells which are built on three lattice vectors **a**_**1**_, **a**_**2**_ and **a**_**3**_. The supercells are oriented in such a way that the (*hkl*) generalized stacking fault plane of interest is normal to the Cartesian *z* direction (Fig. 1). The **a**_**3**_ lattice vectors are thus initially aligned with crystallographic directions [100], [010], [001], [10 1 0] and [012] for (100), (010), (001), (110) and (011) planes, respectively. Thus, for (110) and (011) calculations, monoclinic atomic configurations with angles 90.62° and 92.57° are designed. It should be noted that in complex materials there could be several non-equivalent geometric locations of a slip plane with a given (*hkl*) index which distort different atomic bonds. All possible configurations of slip planes in the post-perovskite structure are thus considered in detail in the Results section. The optimum size of a simulation supercell along the *z* direction is found to be about 100 Å, which is large enough to allow relaxation of atoms close to the slip plane and to prevent the effect of atomic distortions on the boundary condition. Typical orthorhombic simulation supercell contains about 2000 atoms, while a monoclinic supercell contains about 3000 atoms. The excess energies *γ*, corresponding to the energy cost resulting from a rigid-body shear, are calculated by displacing the upper part of a supercell over the lower part across a chosen (*hkl*) slip plane applying a shear displacement *f* = *e*_1_**a**_**1**_ + *e*_2_**a**_**2**_ (Fig. 1). Using a 5 % increment of displacement *e*_i_, the resolution of the energy landscape corresponds to 400 calculation points. In order to keep a periodic boundary condition in an atomic system containing a stacking fault, the lattice vector **a**_**3**_ is given an additional component along the same vector **f** (Fig. 1). After shearing the upper part, atomic relaxations are allowed along the direction perpendicular to the glide plane, i.e., along *z*, exclusively in order to avoid spurious recovery of the perfect crystal geometry during energy minimization at smallest shear step. The energy minimization is performed at constant volume, corresponding to the ground-state volume of a perfect crystal under the given external pressure field.

*γ*-Surface excess energies can be used to evaluate the ideal shear stress (ISS), i.e., the upper limit of the stress that a perfect crystal can sustain (Paxton et al. 1991). ISS is then computed as the absolute maximum of the *γ*-surface energy derivatives relative to the applied shear.

## Results

### Structural parameters and elastic properties

An orthorhombic MgSiO_3_ unit cell is optimized at *P* = 120 GPa and *T* = 0 K using GULP package (Gale and Rohl 2003). The calculated structural parameters and elastic constants are given in Table 2. The *a* and *b* parameters computed with the potential model are slightly overestimated in comparison with experimental data and DFT calculations, while the *c* parameter is a bit underestimated. In general, all structural parameters (including the unit cell volume) are in good agreement with previous experimental and theoretical data since differences do not exceed 3 %.

**Table 2:** Lattice parameters (in Å) and elastic constants (in GPa) for MgSiO_3_ post-perovskite calculated in this study using the potential model by Oganov et al. (2000) with comparison with data from previous studies

This study	*a*	*b*	*c*	*C*_11_	*C*_22_	*C*_33_	*C*_12_	*C*_13_	*C*_23_	*C*_44_	*C*_55_	*C*_66_
	2.521 (1.9–2.7%)	8.124 (0.3–1.0%)	6.050 (0.7–1.4%)	1118 (8.7–14.5%)	781 (15.8–19.3%)	1062 (12.3–18.2%)	509 (14.6–24.4%)	410 (19.5–26.2%)	601 (18.5–25.7%)	286 (3.0–3.2%)	119 (42.8–54.2%)	256 (36.6–41.7%)
GGA^1^	2.474	8.121	6.138	1252	929	1233	414	325	478	277	266	408
LDA^2^	2.455	8.051	6.099	1270	937	1264	425	329	493	291	264	412
LDA^3^	2.462	8.053	6.108	1308	968	1298	444	343	507	295	278	439
GGA^4^	2.474	8.112	6.139	1225	928	1211	409	328	484	281	260	404
Experiment^5^	2.456	8.042	6.093									

Differences with DFT studies are indicated in brackets. Superscripts refer to the following studies: ^1^Oganov and Ono (2004); ^2 ^Iitaka et al. (2004); ^3 ^Tsuchiya et al. (2004a,b); ^4 ^Carrez et al. (2007); ^5 ^Murakami et al. (2004)

The elastic *C*_*ij*_ tensor provided by the potential model (Table 2) is found to be in a reasonable agreement with the available literature data. Generally, all non-diagonal components are about 20 % stiffer, while all diagonal components are about 15 % softer, except *C*_55_ and *C*_66_ which differ by 40–50% from DFT values (Table 2).

### Generalized stacking faults

The *C*-lattice of the post-perovskite results in four potential shear vectors: [100], [010], [001] and ½[110] which correspond to the shortest lattice repeats. For a given shear direction, potential glide planes are expected to be among those with the largest *d*_*hkl*_ distance, i.e., with the lowest Miller index of the (*hkl*) plane. Thus, there are five potential glide planes and ten slip systems to test: [100](010), [100](001), [100](011), [010](100), [010](001), [001](100), [001](010), [001](110), ½[110](001) and ½[${\overline {1}}10$](110).

#### Location of the stacking faults

In contrast to metals, most minerals have complex crystal structures with interatomic bonds of significantly different nature. This fact requires a detailed consideration of bonding and makes the aspect of crystal chemistry to be dominant over the simple concept of close-packed atomic planes for predicting the most probable slip planes. Thus, for each (*hkl*) plane, we consider different possibilities of its location along *z*_hkl_^1^ in order to analyze the effect of different types and number of bonds involved.

**Fig. 2:**
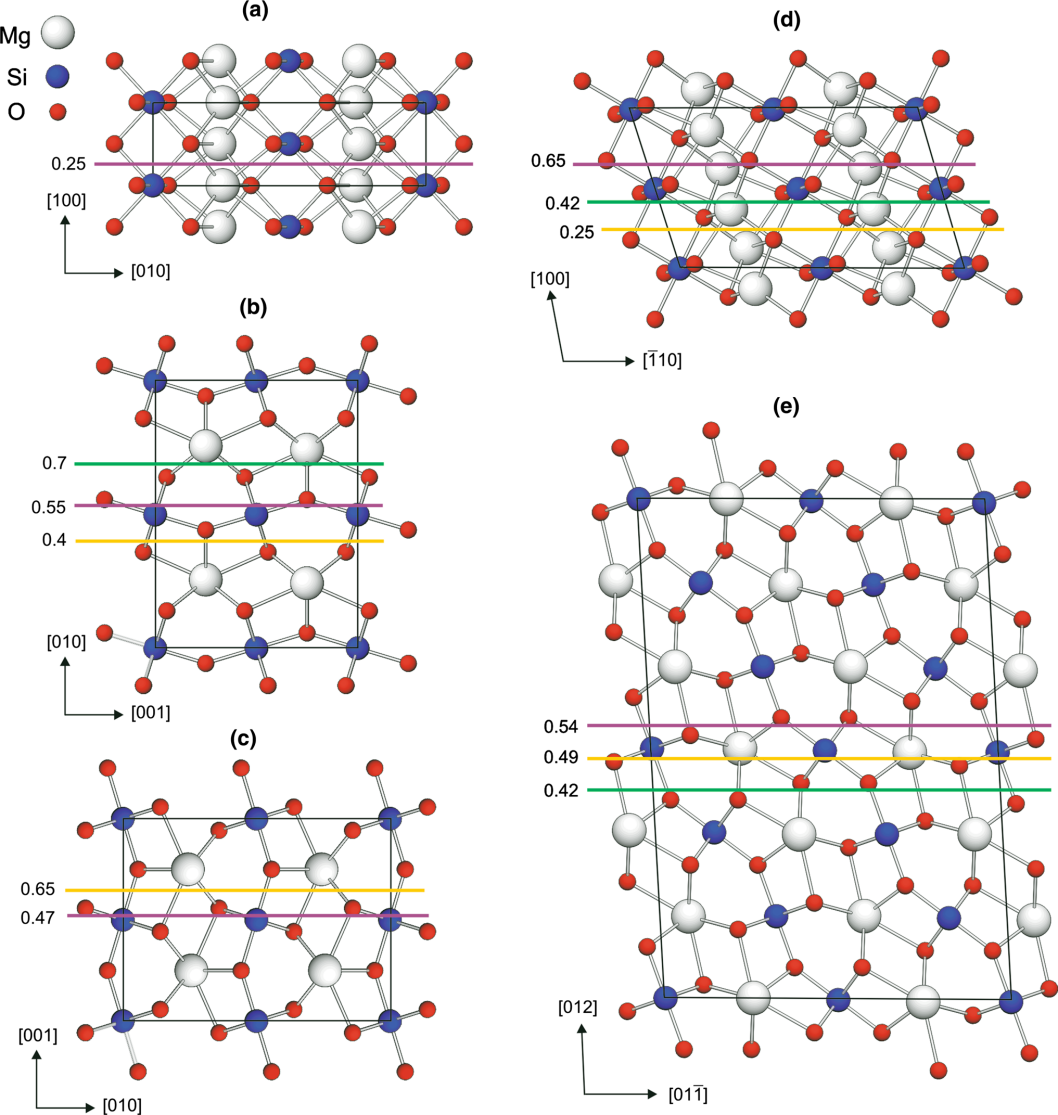
Various non-equivalent geometric locations of slip planes (100) (**a**), (010) (**b**), (001) (**c**), (110) (**d**) and (011) (**e**) in the post-perovskite structure

Atomic planes parallel to (100) are the most densely packed planes in the post-perovskite structure. All atoms in the structure are located at two levels along the shortest [100] direction: z_100_ = 0 and *z*_100_ = 0.5 (Fig. 2a). Consequently, for the (100), there is only one cutting level located at *z*_100_ = 0.25 (equivalent to *z*_100_ = 0.75 according to the symmetry of the structure).

The slip planes (010), (110) and (011) have three non-equivalent potential geometric locations for shear (Fig. 2b, d, e). In the (010) plane (Fig. 2b), shear can be imposed at the levels *z*_010_ = 0.7 (equivalent to 0.2, 0.3 and 0.8), *z*_010_ = 0.4 (equivalent to 0.1, 0.6 and 0.9) and *z*_010_ = 0.55 (equivalent to 0.05, 0.45 and 0.95). For the (110) plane (Fig. 2d), shear can be applied at *z*_110_ = 0.25 (equivalent to 0.75), 0.42 (equivalent to 0.08, 0.58 and 0.92) and 0.65 (equivalent to 0.15, 0.35 and 0.85). The (011) plane (Fig. 2e) can be sheared at *z*_011_ = 0.42, 0.49 and 0.54 (equivalent to ±0.08, ±0.01 and ±0.04, respectively, with periodicity 1/6 (3.64 Å) along *z*_011_ such as in the designed monoclinic cell there are 12 symmetrical replicas for each cutting level). For the (001) glide plane (Fig. 2c) there are two possibilities: *z*_001_ = 0.65 (equivalent to 0.15, 0.35 and 0.85) and 0.47 (equivalent to 0.03, 0.53 and 0.97). The number of the affected bonds per unit cell for each of the listed cutting levels is given in Table 3.

**Table 3:** Characteristic parameters of the investigated slip systems in MgSiO_3_ post-perovskite from *γ*-surface calculations in comparison with previous theoretical studies (^1^Carrez et al. 2007; ^2^Metsue and Tsuchiya 2013)

Slip system	Impacted bonds (per unit cell)	*γ*_max_ (J/m^2^)	*γ*_sf_ (J/m^2^)	ISS (GPa)
[100](010)				
*z*_010_ = 0.55	6 Si–O	6.90		82.5
*z*_010_ = 0.4	2 Mg–O, 4 Si–O	6.37		78.3
*z*_010_ = 0.7	8 Mg–O	4.00		47.9
GGA^1^		5.55		71.7
LDA^2^		6.01		78.1
[100](001)				
*z*_001_ = 0.47	4 Mg–O, 6 Si–O	8.32		102.2
*z*_001_ = 0.65	6 Mg–O, 2 Si–O	3.32		41.0
GGA^1^		4.86		61.8
LDA^2^		5.04		63.4
[100](011)				
*z*_011_ = 0.49	8 Mg–O, 6 Si–O	7.53		93.9
*z*_011_ = 0.54	7 Mg–O, 4 Si–O	4.99		60.5
*z*_011_ = 0.42	6 Mg–O, 2 Si–O	3.33		40.6
GGA^1^		4.91		61.7
LDA^2^		6.31		80.1
[010](100)				
*z*_100_ = 0.25	12 Mg–O, 8 Si–O	30.14		113.3
GGA^1^		21.02		115.8
LDA^2^		19.25		82.4
[010](001)				
*z*_001_ = 0.47	4 Mg–O, 6 Si–O	25.14	4.31	178.1
*z*_001_ = 0.65	6 Mg–O, 2 Si–O	19.33	3.32	137.0
GGA^1^		18.11	4.89	131.2
LDA^2^		17.79	5.04	117.3
[001](100)				
*z*_100_ = 0.25	12 Mg–O, 8 Si–O	13.56	13.23	95.2
GGA^1^		11.66	9.01	107.1
LDA^2^		11.35	7.35	119.0
[001](010)				
*z*_010_ = 0.55	6 Si–O	11.69	6.18	113.0
*z*_010_ = 0.4	2 Mg–O, 4 Si–O	18.32	12.42	173.8
*z*_010_ = 0.7	8 Mg–O	6.29	3.72	68.2
GGA^1^		7.50	4.95	78.0
LDA^2^		7.49	4.56	71.4
[001](110)				
*z*_110_ = 0.65	8 Mg–O, 4 Si–O	10.22	8.15	95.7
*z*_110_ = 0.25	6 Mg–O, 4 Si–O	11.82	11.80	85.7
*z*_110_ = 0.42	6 Mg–O, 4 Si–O	9.44	7.90	83.7
GGA^1^		11.42	10.14	101.6
LDA^2^		11.54	7.42	120.6
½[110](001)				
*z*_001_ = 0.47	4 Mg–O, 6 Si–O	25.43		171.9
*z*_001_ = 0.65	6 Mg–O, 2 Si–O	18.33		126.9
GGA^1^		16.23		120.0
LDA^2^		15.81		120.1
½[${\overline {1}}10$](110)				
*z*_110_ = 0.65	8 Mg–O, 4 Si–O	8.95		82.9
*z*_110_ = 0.25	6 Mg–O, 4 Si–O	14.39		111.9
*z*_110_ = 0.42	6 Mg–O, 4 Si–O	12.54		92.2
GGA^1^		14.44		133.4
LDA^2^		13.65		100.04

*γ*_max_ represents the maximum of the *γ*-line, *γ*_sf_ represents the energy of the stable stacking fault (for *γ*-lines with camel hump shape), ISS represents the ideal shear stress and *z*_hkl_ corresponds to the cutting level along Cartesian *z* direction normal to the (*hkl*) plane

#### GSF excess energies

**Fig. 3:**
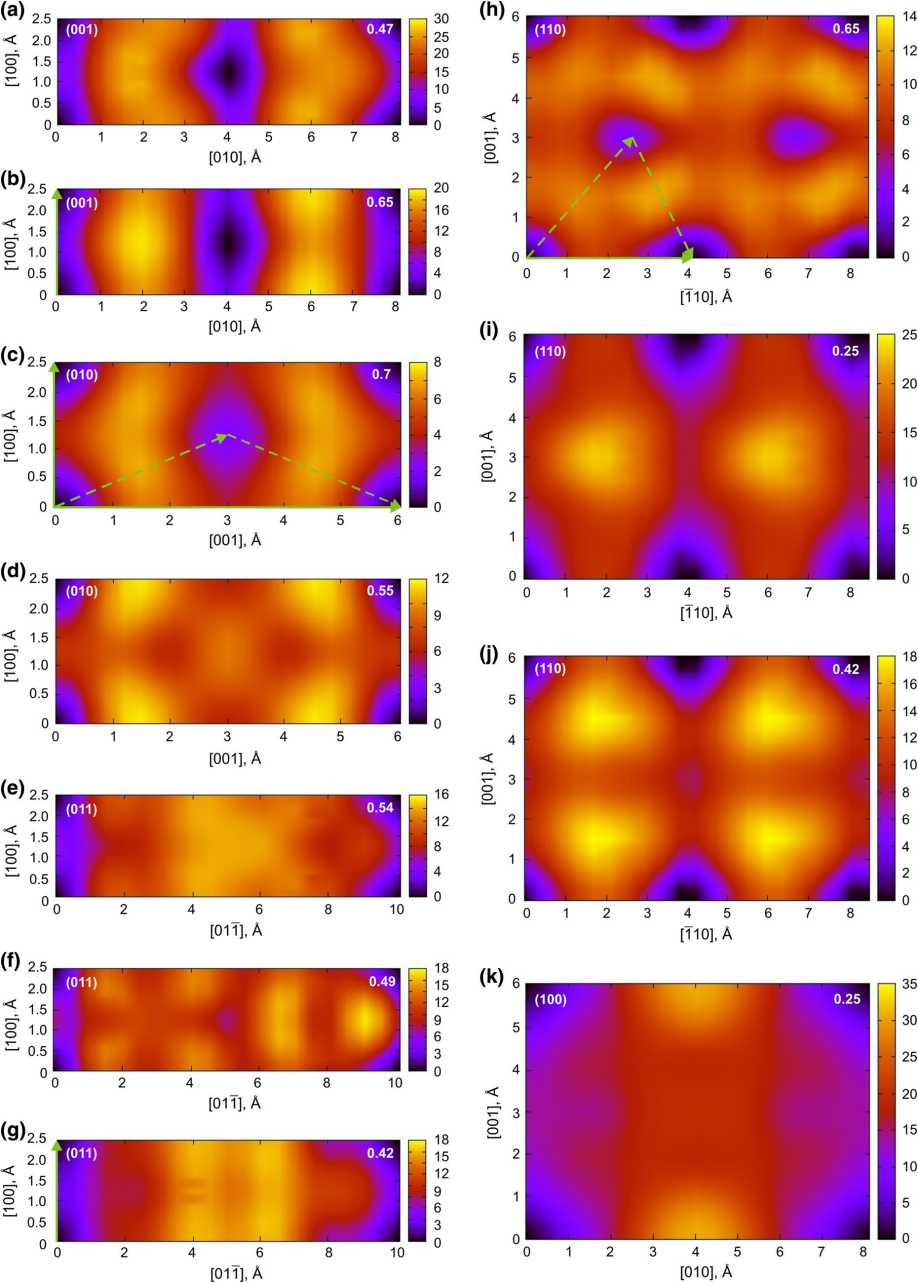
2D projections of *γ*-surfaces (in J/m^2^) computed in this study for (001) (**a**, **b**), (010) (**c**, **d**), (011) (**e**–**g**), (110) (**h**–**j**) and (100) (**k**) slip planes. The corresponding shear levels are given on each plot. Easy slip directions are highlighted with *green arrows*

Previous ab initio studies of GSF in MgSiO_3_ post-perovskite provided only *γ*-lines (Carrez et al. 2007; Metsue and Tsuchiya 2013). In this work, full *γ*-surfaces are calculated for all possible slip planes and cutting levels. The shape of the obtained *γ*-surfaces clearly reflects the symmetry of the structure in a given glide plane for all configurations considered in this work (Fig. 3a–k). In agreement with the *Cmcm* space group, each *γ*-surface exhibits a mirror plane *m* perpendicular to the [100] and [001] directions. Due to the *C*-lattice, ½[110] translation vector is clearly seen in (001) and (110) *γ*-surfaces (Fig. 3a, b, h–j) such as all energy minimum valleys and maximum peaks are translated by ½ <110>. For (010) with *z*_010_ = 0.7 and (110) with *z*_110_ = 0.65 *γ*-surfaces, there are metastable stacking faults (Fig. 3c, h) whose presence is not caused by additional translation vectors of the lattice but by the favorable atomic arrangement in these configurations of faulted crystals. In case of (010), the stacking fault is located exactly in the middle of the *γ*-surface (Fig. 3c) and corresponds to a high-symmetry configuration where atoms are arranged in such a way that slip and unslip halfcrystals represent a mirror reflection of each other (due to the superposition of plane *n*_010_ and ½<101> shear component). For the (110) plane (Fig. 3h), stacking faults are centered with respect to [001] and disposed at 1/3[110] with ½<110> periodicity. Rigid shear by 1/3[110] results in a stacking fault structure where Si-octahedra are connected by corners instead of edges along the slip plane. This geometry reproduces the faulted structure related to post-perovskite–perovskite transformation previously described by (Oganov et al. 2005). Additional shear by ½[001] allows to optimize location of Mg atoms while keeping the same specific arrangement of Si close to the slip plane. Presence of metastable stacking faults in the middle of *γ*-surfaces may indicate simultaneous presence of ½[100] and ½[001] components for dislocations lying on (010) as well as 1/3[110] and ½[001] components for dislocations related to (110) as it is shown with arrows on Fig. 3c, h. Among all examined *γ*-surfaces, the (010) plane with *z*_010_ = 0.7 (Fig. 3c) is characterized by the lowest excess energies, while the highest energy barriers are observed for (100) and (001) at *z*_001_ = 0.47 along the longest [010] direction (Table 3; Fig. 3a, k).

**Fig. 4:**
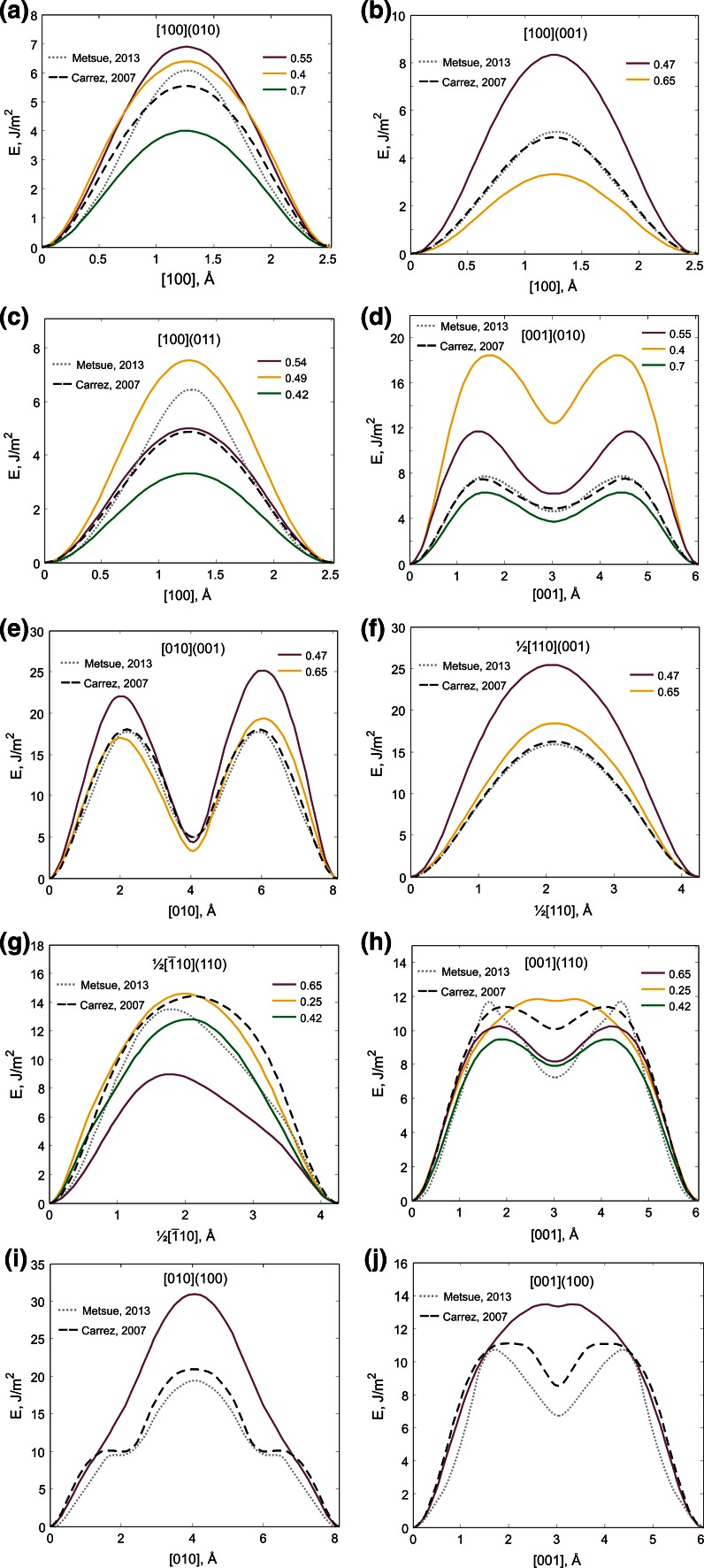
*γ*-Lines for [100](010) (**a**), [100](001) (**b**), [100](011) (**c**), [001](010) (**d**), [010](001) (**e**), ½[110](001) (**f**), ½[${\overline {1}}10$](110) (**g**), [001](110) (**h**), [010](100) (**i**) and [001](100) (**j**) slip systems calculated in this work with pairwise potential model in comparison with previous DFT studies (Carrez et al. 2007; Metsue and Tsuchiya 2013)

Glide along the [100] and ½<110> directions are characterized by a single maximum peak, while glide for all the other systems exhibit a camel hump shape with a local minimum at 50 % along the shear vector (Fig. 4a–j). This indicates the presence of a stable stacking fault suggesting the possibility of dissociation of [001] and [010] Burgers vectors into two partial dislocations with ½[001] and ½[010] Burgers vectors. The base-centered *C*-lattice affects the shape of the [010](001) *γ*-line (Fig. 4e). As noted above, in (001) translation of atoms and, consequently, of GSF excess energy, maximum peaks and minimum valleys occur also by ½[110] (Fig. 3a, b) resulting in asymmetrical shapes of [010](001) *γ*-lines. Both for z_001_ = 0.65 and z_001_ = 0.47, the maximum peaks located at ¾[010] are about 2.5 J/m^2^ (10–12 %) higher than peaks at ¼[010] which can be explained by differences in geometry of faulted crystals with ¼[010] and ¾[010] rigid shear. Thus, close to the slip plane, configuration with ¾[010] shear is characterized by shorter Mg–Si distances (by ~0.5 Å) and longer Si–O bonds with apical oxygen (by ~0.4 Å) than those with ¼[010] shear. However, it should be noted that the observed asymmetric shapes are not so distinct for the DFT curves (Carrez et al. 2007; Metsue and Tsuchiya 2013). The lowest energy barriers are related to three slip systems which involve the smallest shear vector [100] (*b* = 2.521 Å) and to the [001](010) slip system with *z*_010_ = 0.7 (only Mg–O bonds are impacted). The highest energy barrier corresponds to the [010](100) system (Table 3).

## Discussion

### Validity of the empirical potential

Considering the close lattice parameters obtained from interatomic potential with DFT and comparable results in major compressional and shear elastic constants (Table 2), we feel this set of parameters has the level of transferability from Pv to Ppv, although we do expect the differences between force field calculations and density functional calculations, since an insurmountable task is in practice to use simplified classical mechanics model to simulate quantum systems on bond formation energies and bond geometry. However, modeling plasticity and defects requires transferability of the potential to reproduce atomic configurations far from equilibrium where the potential is fitted. In order to validate the chosen parameterization for dislocation modeling at atomic scale, its ability to reproduce ab initio calculations of *γ*-lines is tested (Zimmerman et al. 2000; Godet et al. 2003; Ryu et al. 2009; Jelinek et al. 2012; Pizzagalli et al. 2013).

The *γ*-surface energies computed in this work for the (010), (001), (011) and (110) glide planes are in good agreement with previous ab initio simulations (Carrez et al. 2007; Metsue and Tsuchiya 2013) (Table 3; Fig. 4a–h). All DFT *γ*-lines for (010) and (001) are in agreement with those obtained with the semi-empirical potential at cutting levels leading to the lowest energy barriers, i.e., to *z*_010_ = 0.7 and *z*_001_ = 0.65, respectively (Fig. 4a, b, d–f). Discrepancies observed for [100](010) and [100](001) slip systems may be attributed to either the underestimation of *C*_55_ and *C*_66_ elastic coefficients predicted by the potential model (Table 2) or to an overestimation of excess energies in DFT calculations resulting from a size effect of the volume used in DFT approach. For (011) and (110), *γ*-lines from DFT correspond to different cutting levels. Thus, the [100](011) *γ*-line by Carrez et al. (2007) is related to *z*_011_ = 0.42, while the *γ*-line by Metsue and Tsuchiya (2013) reproduces the shape of the high-energy barrier obtained with *z*_011_ = 0.49 (Fig. 4c). Considering *γ*-lines for (110), the ½[${\overline {1}}10$](110) asymmetric curve by Metsue and Tsuchiya (2013) is consistent with our lowest energy barrier obtained with *z*_110_ = 0.65, while results by Carrez et al. (2007) agree with the high-energy symmetric curve obtained at *z*_110_ = 0.42 (Fig. 4g). However, the easiest cutting level is not the same for ½[${\overline {1}}10$] and [001] directions: along [001], the lowest energy barrier in (110) corresponds to *z*_110_ = 0.42 (Fig. 4h). This phenomenon is considered below in detail.

For (100), the potential model leads to *γ*-lines with systematically higher energies and comparatively dissimilar shape (Table 3; Fig. 4i, j). The stacking fault along [001](100) which appears in DFT curves is barely visible with the potential (Fig. 4j). Similarly, along [010](010), the shoulders at 25 % of shear found with the DFT are not reproduced by the potential (Fig. 4i). However, both DFT and potential model clearly show that (100) is one the most unfavorable planes for shear which involves very high-energy configurations.

Apart from the discrepancy observed for very unfavorable shear planes like (100), results on the *γ*-surfaces obtained from the potential model compare well with ab initio calculations. The empirical parameterization of pairwise potentials proposed by Oganov et al. (2000) is thus valid to model shear properties of post-perovskite and in particular crystal defects modeling involving atomic positions far from equilibrium.

### Influence of crystal chemistry

Analyzing the computed *γ*-lines from different cutting levels for each (*hkl*) plane, it is found that the lowest and the highest energy barriers do not necessarily correspond to the same cutting levels for shear along different directions within the same plane. For instance, in the (010) plane, shear at z_010_ = 0.7 cuts only Mg–O bonds (8 bonds per unit cell), whereas only Si–O bonds (6 per unit cell) are cut at z_010_ = 0.55 and both Mg–O and Si–O bonds (2 Mg–O and 4 Si–O per unit cell) are cut at z_010_ = 0.4. While the lowest excess energy corresponds to z_010_ = 0.7 for shear along [100] and [001] in (010), the highest energy barriers correspond to different cutting levels (Fig. 4a, d). In (010), shear is easier at z_010_ = 0.55 compared to z_010_ = 0.4 along [001], whereas along [100], the energy barrier is higher at z_010_ = 0.55, although the difference with z_010_ = 0.4 is very small (Fig. 4a, d; Table 3). A similar effect is observed for shear along ½[${\overline {1}}10$] and [001] in (110) with cutting levels 0.42 (6 Mg–O and 4 Si–O) and 0.65 (8 Mg–O and 4 Si–O). But in this case, the easiest cutting level is not the same for both directions (Fig. 4g, h; Table 3). This behavior illustrates that the number and nature of bonds is not enough to describe the easiness of shear. The ability for building up new bonds after shear is likely to play an important role.

### Searching for the easiest slip systems

In order to further describe the relative resistance to shear of the potential slip systems, the ideal shear stresses (ISS) are calculated (Table 3). ISS represents the upper limit of stress that a perfect crystal can sustain before yielding (Paxton et al. 1991). The lowest ISS values together with the lowest *γ*-surface excess energies can point to the easiest slip systems, eventually contributing to the development of deformation textures. The smallest ISS values, as well as the lowest *γ*-energies, correspond to the slip systems with the shortest [100] shear vector and to the [001](010) slip system with the glide plane located at the level 0.7 along the [010] direction (Table 3). In order to analyze an effect of the reduced *C*_55_ and *C*_66_ values predicted by the potential model on the calculated ISS for these slip systems, the ISS values can be normalized by the corresponding *C*_*ij*_ components and compared with those from DFT. Both DFT and semi-empirical values provide the same ISS/*C*_*ij*_ ratios (0.25, 0.3 and 0.19 for [001](010), [100](001) and [100](010), respectively). This correlation between computed elastic constants and ISS indicates that underestimated *C*_55_ and *C*_66_ values inevitably result in smaller ISS values (~35%) for [100] slip systems predicted by the potential model (Table 3). However, even keeping in mind such an effect of reduced ISS for [100] systems, they clearly remain among the most probable slip systems together with [001](010) what is consistent with the ab initio studies by Carrez et al. (2007) and Metsue and Tsuchiya (2013). The empirical potential model also shows that ½[${\overline {1}}10$](110) (at z_110_ = 0.65) and [001](110) (at z_110_ = 0.42) are further possible slip systems. While considering all possible cutting levels in the post-perovskite structure, we show that the lowest and the highest energy barrier can correspond to different shear levels for different directions within the same glide plane. This effect was not taken into account in previous ab initio studies and therefore has affected the (011) and (110) *γ*-surface calculations. Possible relevance of the ½[${\overline {1}}10$](110) system is in agreement with slip predicted by Oganov et al. (2005) based on first-principle metadynamics.

### Implications

In MgSiO_3_ post-perovskite, plastic anisotropy represents a potential cause for seismic anisotropy in the D″ layer through the development of crystal-preferred orientations (CPO) during plastic flow. Experimental CPO produced in the diamond anvil cell with MgSiO_3_ post-perovskite and its close structural analog MgGeO_3_ yield conflicting results. According to Merkel et al. (2006, 2007), both MgSiO_3_ and MgGeO_3_ post-perovskite phases exhibit slip on (110) and/or (100). High-pressure experiments on the same phases by Miyagi et al. (2010; 2011) reveal strong (001) texture development during the perovskite → post-perovskite transformation and subsequent slip on (001) in the [100] direction in the transformed post-perovskite phase. However, the present study rather emphasizes the role of slip in (010). Dislocation modeling will be performed using the potential validated here to further constrain the easy slip systems in MgSiO_3_ post-perovskite.

## Conclusions

An effective empirical parameterization of pairwise potentials (Oganov et al. 2000) developed for MgSiO_3_ perovskite (bridgmanite) was applied to the next high-pressure phase—post-perovskite. We have examined its transferability to reproduce the structure, elastic properties and *γ*-surface excess energies of post-perovskite. The significant structural, elastic and energetic parameters computed with the potential model were shown to compare well with available theoretical and experimental data.

Calculations of the *γ*-surface energies were performed taking into account all possible non-equivalent geometric locations of slip planes breaking different bonds in the post-perovskite structure. The observed phenomenon of switching the cutting level with lowest energy barrier for different slip directions within one glide plane (see [001](110) and ½[${\overline {1}}10$](110) systems) highlights the necessity to consider all possible cutting levels for complex materials. In agreement with previous ab initio studies (Carrez et al. 2007; Metsue and Tsuchiya 2013), the slip systems involving the shortest [100] shear vector and the [001](010) system within the glide plane cutting only Mg–O bonds are characterized with very low *γ*-surface energies. Based on current *γ*-surface calculations, (110) could be considered as further possible slip plane.

Reasonable agreement with previous theoretical and experimental results proves the chosen potential parameterization (Oganov et al. 2000) to be valid for further fully atomistic modeling of dislocations and their mobility in MgSiO_3_ post-perovskite.

## Footnotes

1 Here and hereafter, *z*_hkl_ corresponds to the Cartesian *z* normal to the (*hkl*) plane.
